# Delirium care in hospitals in Ireland on World Delirium Awareness Day 2023

**DOI:** 10.1007/s11845-024-03692-8

**Published:** 2024-05-31

**Authors:** Zahra Azizi, Niamh O’Regan, Tim Dukelow, Teresa Bohane, Eithne Harkin, Christina Donnellan, Ida Carroll, Maria Costello, Susan O’Reilly, Claire Noonan, Erica Walsh, Suzanne Timmons

**Affiliations:** 1 Centre for Gerontology and Rehabilitation, School of Medicine, University College Cork, Cork, Ireland; 2 Department of Geriatric Medicine, Waterford Integrated Care for Older People, University Hospital Waterford, Waterford, Ireland; 3 Cork University Hospital, Cork, Ireland; 4 Bantry General Hospital, Bantry, Ireland; 5 Wexford General Hospital, Wexford, Ireland; 6 Tipperary University Hospital, Clonmel, Ireland; 7 Department of Ageing and Therapeutics, University Hospital Limerick, Limerick, Ireland; 8 Department of Geriatric and Stroke Medicine, University Hospital Galway, Galway, Ireland; 9 Connolly Hospital Blanchardstown, Dublin, Ireland; 10 Tallaght University Hospital, Dublin, Ireland; 11 St Luke’s General Hospital Kilkenny, Kilkenny, Ireland

**Keywords:** Assessment, Awareness raising, Delirium, Education, Practice, Prevention

## Abstract

**Background:**

Acute, transient, but sometimes persistent, delirium is characterized by a sharp disruption in attention, consciousness, and cognitive function, and can be caused by many medications and disorders. Delirium occurrence and negative consequences, such as falls and functional decline, can be decreased with multifactorial prevention and timely detection.

**Aims:**

To describe current clinical practice in relation to the prevention, assessment, and management of delirium in Irish hospitals; awareness-raising and educational activities; and barriers to good practice.

**Methods:**

On World Delirium Awareness Day (15th March 2023), a global survey was conducted of delirium prevalence and care. A senior clinical staff member on each participating ward reported on delirium prevalence at 8AM and 8PM, and on usual ward practice; this data was entered into an online survey by a data collector (typically a clinician from the site, visiting several wards to record data). This study reports data from Irish hospitals.

**Results:**

In total, 132 wards from 15 hospitals across Ireland participated. Almost 60% of wards used ‘personal judgment’ for delirium assessment. Having at least one delirium training session in the preceding year was associated with greater use of a formal assessment tool (60.3% versus 18.8%; p < 0.001). Wards reported staff training/education as the main priority to improve care, but 72.7% of wards identified insufficient time to train staff as a key barrier.

**Conclusions:**

Clinical practice related to delirium care requires improvement. Awareness raising and staff training require more focus and time in busy clinical settings.

## Background

Delirium is a complex neuropsychiatric disorder with sudden onset and variable progression that occurs in all medical settings and affects around 15–20% of general hospital admissions [1]. Delirium is independently associated with several adverse outcomes, with higher morbidity and mortality rates, increased ICU and hospital length of stay, more frequent need for nursing home care following discharge, and increased risk of long-term cognitive impairment [2–8]. Delirium is associated with a five-fold risk of death, and twice the rate of admission to residential care following discharge, and is associated with post-traumatic stress disorder-like symptoms [9–11].

Factors associated with poorer patient prognosis include increased patient age and lengthened delirium duration [12]; with hypoactive delirium being most often missed and having worse mortality [12, 13]. Alongside the debilitating effects of delirium on patients, it presents a significant financial cost to health and social care services, with an estimated additional in-patient cost for a delirium episode ranging between 806 and 24,509 USD, due to increased care costs and length of stay [14]. In the United States, direct one-year costs associated with delirium are 143–152 billion USD, assuming delirium occurs in 20% of patients hospitalised annually [15].

In Ireland’s second round of the National Audit of Dementia Care (INAD-2; data collection in 2019), despite their very high risk of delirium, only 19% of patients with known dementia received delirium screening during a hospital admission, a decrease from 29.7% in 2013 [11]. Accordingly, the Health Service Executive updated and re-published an existing national delirium algorithm (March 2020), recommending that patients over 65 in ED or acute medical assessment units (AMAUs) should be screened for delirium using the 4AT assessment tool [16]. Concurrently, an existing ward delirium algorithm was updated, requiring that patients transferred to medical or surgical wards should have the 4AT conducted on admission if missed in ED or performed over 48 h previously. Both algorithms suggest delirium prevention measures, and investigation and management of delirium, and were circulated to every hospital in Ireland, along with the individual hospital/ hospital group audit performance data, in 2020/2021. Of note, these algorithms align with the National Institute for Health and Care Excellence guideline for delirium prevention and screening [17] (updated 2023) and the Scottish SIGN guideline [18].

On World Delirium Awareness Day (i.e. 15th March 2023), a global survey aimed to describe delirium prevalence, and ward practice for prevention, assessment, and management of delirium, and to identify any barriers to best practice (Start | wdad-study.center). This study reports data from Irish hospitals within this global survey, 2.5 years after the INAD-2 findings and recommendations were publicly launched, in order to gain insight into current practice and barriers, across a wider patient population than just patients with dementia.

## Methods

The study was an observational cohort study on World Delirium Awareness Day in multiple clinical sites in Ireland, to assess the usual practice of delirium screening, assessment, and management, such as delirium assessment tool use, the presence of delirium protocols, awareness raising interventions, delirium related structures and processes, pharmacological management, and others. The global study received ethics approval from Kiel University, Germany (AZ_D 519/22_Aug 22). Ethics approval was granted for secondary data analysis of Irish data by the Clinical Research Ethics Committee of the Cork Teaching Hospitals (ECM 4 (n) 13/12/2022).

### Participants

Patients were investigated indirectly in clinical settings in the Republic of Ireland, including acute hospitals, and residential care units and in-patient rehabilitation units within acute or community hospital campuses. Instead of obtaining direct patient data, ward data was pooled by the manager on each ward. The survey respondents (i.e. participants) were clinical or research workers who worked as data collectors, visiting each ward in turn.

### Data collection and sharing

Hospital data included a) number of beds, b) affiliation (e.g. university-linked, private, etc.). Unit/ward-based data included a) patient age category (18–75; over 75; or mixed); b) ward main discipline (e.g. medical/surgical, etc.); c) ward type (e.g. ICU, general ward etc.); d) number of beds; and e) presence/absence of written protocols for pain management, dementia, and others. Delirium-specific data on the ward/unit included a) delirium awareness raising interventions for staff, such as training, posters, and others; b) delirium assessments performed (multiple formal tools listed in the survey, with options for “none”, “other tool” and “personal judgement”); c) frequency of this delirium assessment; and d) profession primarily responsible for assessment (e.g., nurses, occupational therapists, etc.). Data on prevention and treatment interventions were collected. In this study, a prevention measure was counted as present if > 50% of patients on the unit/ward received it as a routine intervention, at least once per shift. The same criteria applied for management interventions (i.e. > 50% of people with delirium received them), separated into non-pharmacological and pharmacological interventions. Barriers to implementation and/or use of evidence-based strategies regarding delirium management were also collected.

The manager for each ward/unit reported verbally on usual ward processes relating to delirium to the data collectors (survey participants) for each site, in early March 2023. The data collectors arranged a convenient time to collect data from the ward manager and recorded this on a standardized paper-based collection sheet, comprising thirty-four questions, where all but three involved quantitative data, and seven had options for additional information. The survey had been pretested with clinicians for understanding, feasibility, and time required.

The lead clinician in each site stored this sheet in a secure location (noting that it did not contain any personal data at any time), until it was entered into the global online survey, by midnight, Central European Time, on 18th March 2023. A separate survey was completed per ward. There were no mandatory questions. Participants were asked for their opinions of priorities in a) delirium care and b) delirium research, and for any further comments. The Irish data was retrieved separately using a pre-determined code that all data collectors used. This data will be stored for 10 years after the completion of the study.

### Data analysis

We used the R statistical programming environment, version 4.2.0 [19] for all analyses. Nominal data were reported in relative extents as percentages; the numerator/denominator value was also reported whenever this was less than 90% of the total data set. To compare categorical data between distinct groups, chi-square tests were performed. Wilcoxon signed (for two groups) and Kruskal–Wallis (more than two groups) tests were used to compare continuous variables across groups. We performed a regression analysis relating to using a formal delirium tool, as dependent variable, with the presence of delirium-related structures and processes as predictors, by applying generalized logistic mixed-effect models by “glmer” in the “lme4” package (family = “binomial”) [20]. The level of significance was established as 95% (p < 0.05). For two open-text questions, data analysis followed a content analysis approach [21].

## Results

### Demographics

This research included 132 wards from 15 hospitals across the Republic of Ireland, with the South-Southwest hospital group predominating (n = 88 wards), and the north-west, north-east and midlands not represented. The majority (92.4%) were university-affiliated, with four being rehabilitation or long-term care units. The size of the participating hospitals varied, with 32.6% and 29.5% of participating wards located in hospitals with 500–750 beds and 250–500 beds, respectively, while 20.5% were located in hospitals with < 250 beds and 17.4% in hospitals with > 750 beds.

The participating wards were diverse, with the majority being medical or combination medical-surgical (n = 54; including one oncology ward), geriatric wards (n = 20), and intensive care units (n = 21). There were fewer EDs or AMAUs (together as ‘ED/AMAU’; n = 9) and surgical wards (n = 15). There were just a few long-term care wards (n = 7), rehabilitation wards (n = 5), and transitional care (step-down) wards (n = 1); they are combined together as “non-acute wards” in analyses.

Most informants for the ward data were nurses (47%), followed by physicians (26.5%) and managers (18.2%). These had a lead or partial-lead position in the ward in 94.6% of cases. Mostly, these informants had < 5 or 5–10 years’ experience (31.1% and 28% respectively) on the ward/unit, while 18.9% had more than 20 years’ experience, 12.9% had 10–15 years’ experience, and 9.1% had 15–20 years’ experience.

**Fig. 1:**
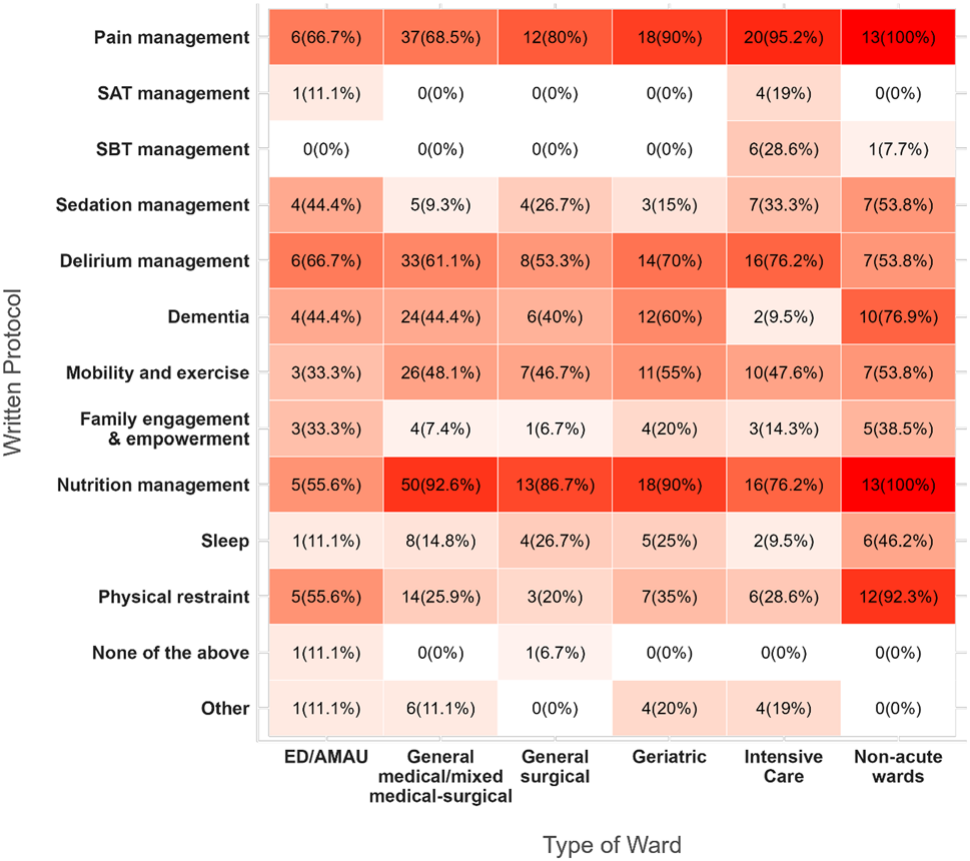
The presence of written protocols, across different ward types. Darker shades represent higher levels. *SAT: Spontaneous Awakening Trials, SBT: Spontaneous Breathing Trials*

**Fig. 2:**
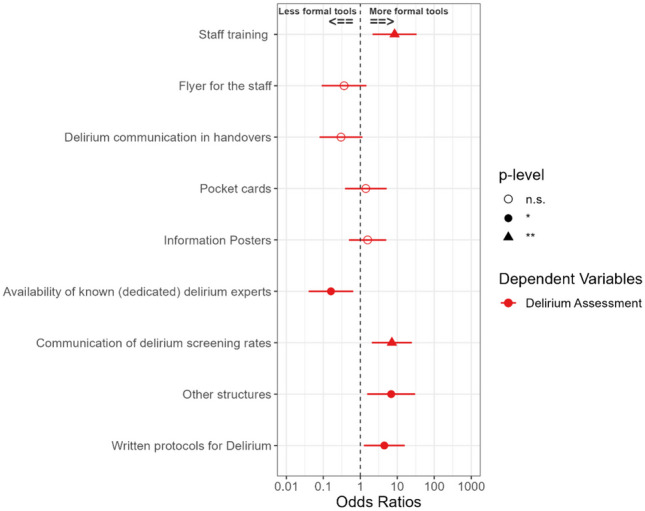
Odds ratio (OR) for using a formal tool for delirium assessment, based on predictors, with 95% confidence intervals (n = 132). *p ≤ 0.05; **p ≤ 0.01

**Fig. 3:**
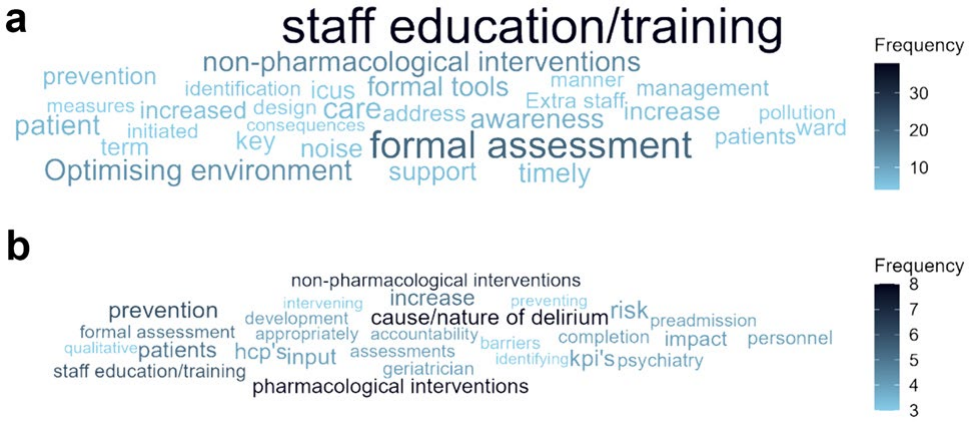
Word clouds for **(a)** priorities for delirium care and **(b)** priorities for delirium research

### Written protocols for delirium-related care

**Fig. 4:**
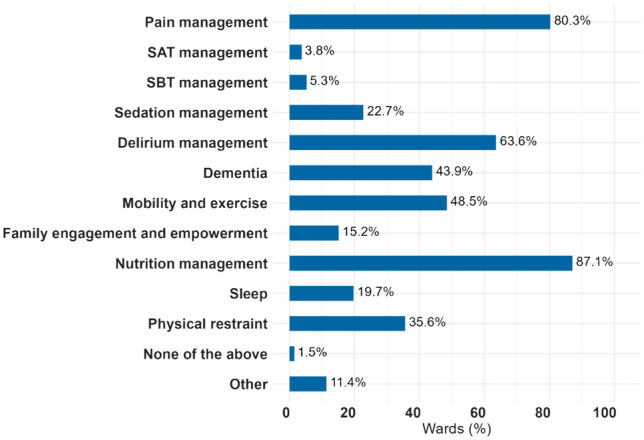
The presence of written protocols relevant to delirium. *SAT: Spontaneous Awakening Trials, SBT: Spontaneous Breathing Trials*

This study investigated the presence of written protocols for delirium and delirium-relevant conditions, Fig. 1. While the presence of a written protocol for pain management (80.3%) and nutrition (87.1%) was generally high, few wards had a written protocol for sleep (19.7%), mobility, family engagement and empowerment. (For more information, please see Fig. 4 for all data). In addition, 19% and 28.6% of the 21 ICU wards had a protocol for spontaneous awakening trials and spontaneous breathing trials, respectively. A protocol for delirium management was reported in 63.6% of wards. Intensive care units (76.2%), geriatric wards (70%), and ED/AMAU (66.7%) had the highest rate of written protocols for delirium, followed by general medical/mixed medical-surgical wards (61.1%) (Fig. 1).

### Delirium-related structures and processes

**Fig. 5:**
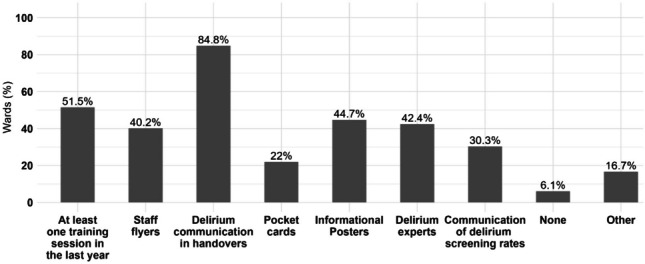
Delirium-related processes and activities. *Note:* Other activities included using the HSEland (i.e. Health Service Executive's national online learning and development portal) (16/21), Delirium champions (2/21), visual guides, orientation devices, and single rooms

**Fig. 6:**
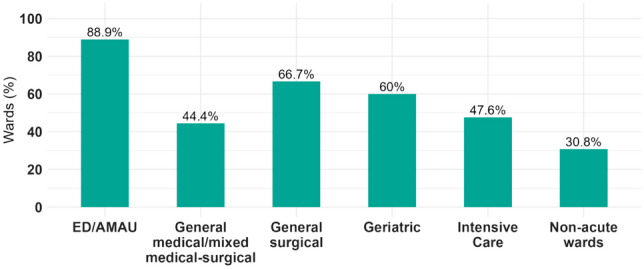
Delirium-related training in the past year, across different types of wards

Processes and structures that might facilitate delirium care included staff training (having at least one delirium educational training session in the preceding year), reminders (staff flyers, pocket cards, information posters), patients’ delirium status being part of handover communication, availability of known (dedicated) delirium experts, and communication (feedback to staff) of delirium screening rates on the unit/ward. Within these, the most frequent activity was delirium communication in handovers (84.8%). Staff training was reported in 51.5% of wards overall (Fig. 5), highest in ED/AMAU (88.9%), followed by general surgical (66.7%), and geriatrics wards (60%) (Fig. 6). In contrast, only 30.8% (4/13) of non-acute wards had provided staff training in the preceding year.

The overall number of these activities did not differ significantly between ward types (Kruskal–Wallis test: H (5, n = 132) = 9.09, p = 0.08), with Emergency Departments/AMAUs utilizing the highest number (Mean = 5, SD = 2.3), then geriatric wards (Mean = 4.2, SD = 2.3), and non-acute wards the least (Mean = 3.5, SD = 1.7).

### Delirium screening and assessment

In total, 59.8% of wards used ‘personal judgment’ for delirium assessment, while 26.5% used 4AT, and a few used Confusion Assessment Method for the ICU (CAM-ICU; 7.6%), Nursing Delirium Screening Scale (NU-DESC; 0.8%) or any other formal tools (5.3%). Staff training in delirium having taken place in the previous year was associated with higher rates of using a formal tool for delirium assessment, at 18.8% in wards without training (12/64) and 60.3% in wards with training (41/68) (χ^2^ = 22.0, *p* < 0.001; n = 132). The association between using a formal delirium assessment tool and other delirium-related processes and activities is reported in Fig. 2 (also in Table 1). There was a positive association between using a formal tool and staff training; communication of delirium screening rates; and written delirium protocols (for Odds Ratios and other details please see Table 1). However, having a delirium expert available had a negative association, while staff reminders (flyers, pocket cards, and posters) or handover communication had no significant effect on using a formal tool to assess delirium.

**Fig. 7:**
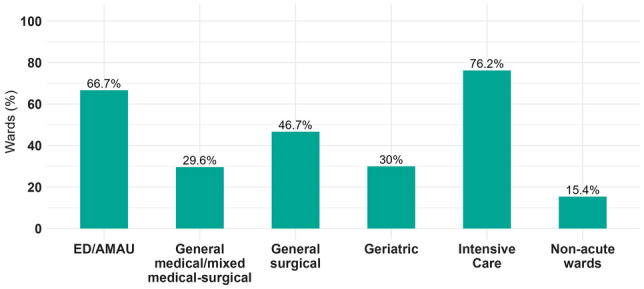
Using a formal assessment tool, across different types of wards

The use of formal delirium assessment tools also varied by ward type, ranging from 76.2% (16/21) and 66.7% (6/9) in intensive care units and ED/AMAU, respectively, to 15.4% (2/13) in non-acute wards (Fig. 7). Using a formal assessment for delirium was not different in wards when the participant reported that the majority (> 75%) of patients were > 75 years (30.6%; 11/36) compared to other wards (44.2%; 42/95) (χ2 = 1.5, *p* = 0.22; n = 132).

**Fig. 8:**
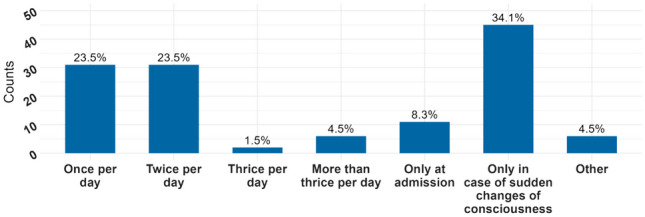
The frequency of delirium assessment (by any means)

While delirium was assessed once or twice per day in almost half of wards (each 23.5%), in more than one in three wards, delirium was assessed only in case of sudden changes of consciousness (Fig. 8).

### Non-pharmacological prevention and treatment

**Fig. 9:**
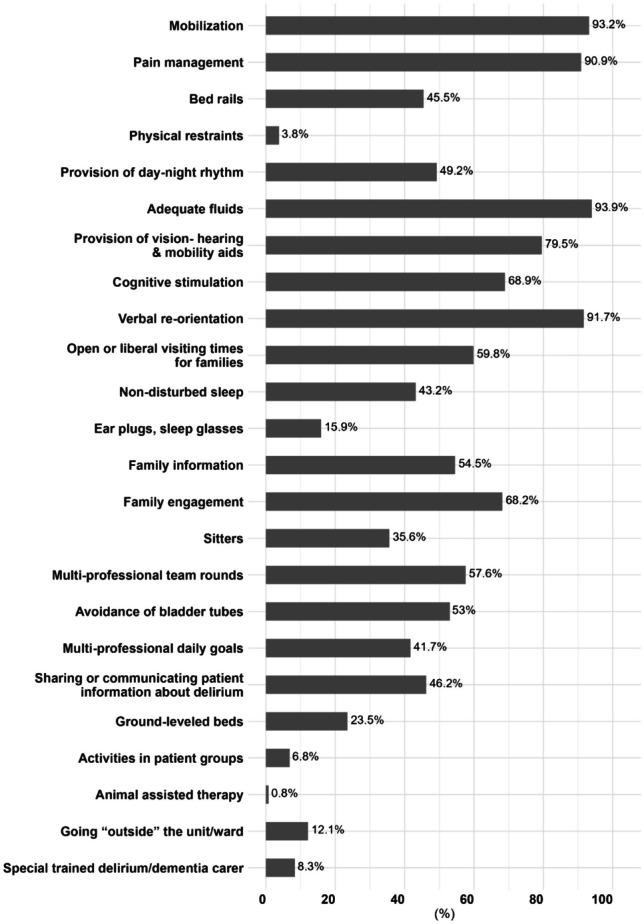
The percentage of wards using a non-pharmacological delirium prevention and management activity

Participants were asked about ways in which most patients (> 50%) on the unit/ward receive routine non-pharmacological interventions (at least once per shift) for delirium prevention and treatment. Interventions such as mobilization (sitting on the edge of the bed, or more, during the day), pain management, adequate fluids, and verbal re-orientation were performed regularly, with over 98% of wards performing at least one of these. Of concern, bed rails (45.5%) and physical restraints (3.8%) were used in some wards (Fig. 9).

**Fig. 10:**
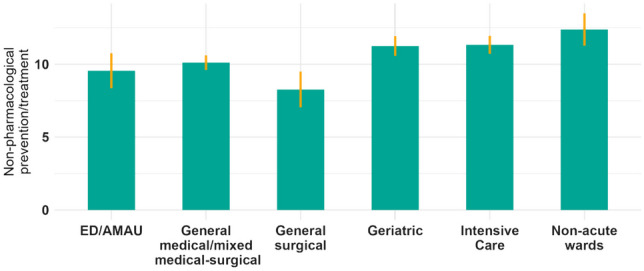
Number of non-pharmacological delirium prevention and management interventions used in wards, across the various ward types. Orange lines correspond to the standard error of the Mean

The number of non-pharmacological delirium prevention and management interventions implemented in wards was calculated (counting the two forms of restraints as negative scores). There was not any significant difference between ward types (Fig. 10; Kruskal–Wallis test: H (5, n = 132) = 9.34, *p* = 0.09), or between wards with staff training in the last year compared to others (Wilcoxon signed rank test W = 1823, *p* = 0.10).

### Pharmacological treatment

The study asked about how, generally, the pharmacological management of delirium on the ward/unit was approached. Almost 70% of wards reported an individualised approach, depending on the patient and the medication side effects (versus a blanket approach), and 72% of wards reported that it depends on the patient’s specific delirium symptoms. In 72.7% of wards, the pharmacological treatment was recorded in handovers; reported discussion with ‘most’ families and patients was poor (50% and 18.9% respectively).

**Fig. 11:**
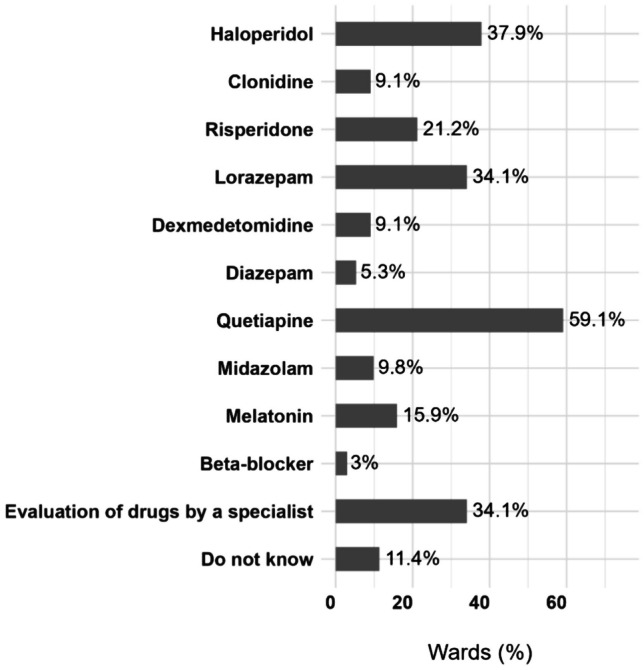
Percentage of wards using different pharmacological treatments for delirium

In total, in 59.1% of wards, the participants stated that most delirious patients (> 50%) receive quetiapine. Other common prescriptions included haloperidol (37.9%) and lorazepam (34.1%). In almost 34% of wards, the participant reported that a specialist (e.g., geriatrician, pharmacist, etc.) evaluated the medications (Fig. 11).

### Barriers

**Fig. 12:**
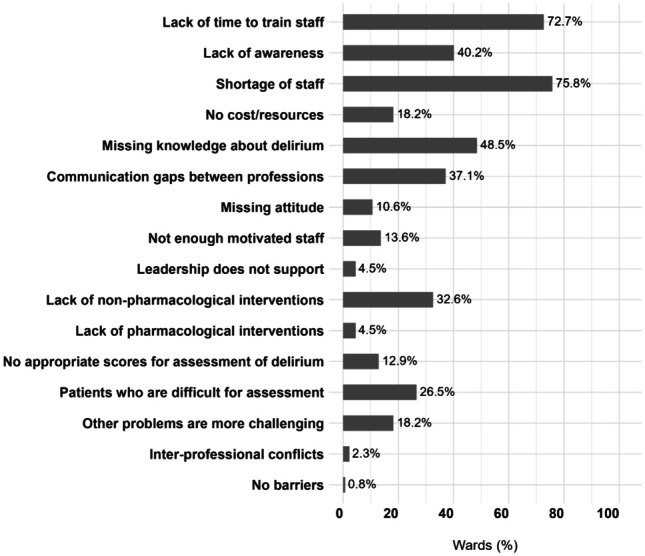
Barriers reported to implementation and/or use of evidence-based strategies in delirium care

Participants selected a wide range of proffered barriers to the implementation and/or use of evidence-based strategies. The most common were a shortage of personnel/staff (75.8%) and lack of time to educate and train staff (72.7%), followed by inadequate knowledge about delirium (48.5%), lack of awareness (40.2%), communication gaps between professions (37.1%), lack of non-pharmacological interventions (32.2%), and patients who are difficult to assess (dementia, dying) (26.5%). Less often selected barriers were having no budget/resources for promoting delirium (awareness), staff attitudes that delirium is not important, no appropriate tools for assessment, or unsupportive leadership (2.3% to 18.3% of respondents). More information can be found in Fig. 12. Of note, one ward did not select any barriers (which may reflect that no barriers exist, or that the participant didn’t explore this with ward staff), while 80% of wards selected more than one barrier. Other barriers suggested by the ward informant (i.e., not offered within the options) included the busy and overcrowded environment (9/24 wards), inexperienced staff and staff turnover (5/24 wards), and lack of time to screen delirium or spend time with patients (5/24 wards).

Participants were asked about their future priorities for delirium care. Based on the thematic analysis of this open question, overall, wards considered training/educating staff to be a priority, such as: “*increased awareness and education of delirium care for all healthcare professionals”.* The need for formal assessments, non-pharmacological interventions, and optimizing the environment for delirium care were also prioritised (Fig. 3a). One participant stated, “*Prevention is key. Non-pharmaceutical measures initiated in a timely manner …. Address the design of ICUs and noise pollution in the environment*.”

Participants’ priorities for delirium research were pharmacological and non-pharmacological interventions, followed by exploring the cause and nature of delirium, prevention, and staff training (Fig. 3b). Of note, while pharmacological intervention was not a priority for delirium care (Fig. 3a), some wards considered this for future research, along with non-pharmacological intervention.

## Discussion

In this study, data was collected as part of a worldwide study on World Delirium Awareness Day on March 15th, 2023. This is the first Irish multicentre study to assess the implementation of delirium processes and activities, and current barriers to delirium care in hospital wards. The findings show that almost three out of five wards used ‘personal judgment’ for delirium assessment (known to miss cases [22]). Staff-focused activities on wards, such as training and providing feedback on delirium screening rates, were associated with greater use of formal assessment tools (although causality cannot be assumed).

ED/AMAU units generally performed better in delirium care compared to other wards, with the highest reported delirium training (88.9%) and second best at using a formal assessment tool (66.7%). Moreover, the number of implemented delirium-related processes and activities was high in ED/AMAU, and the presence of written protocols (66.7%) was among the highest (noting the presence of the national-specific ED/AMAU delirium algorithm; [16]). In contrast, general medical wards (representing 41% of included wards and 46% of patients) had a low rate of staff training, and a low rate of utilizing a formal delirium assessment tool (30%), even though a general medical/surgical ward delirium algorithm also exists.

Training/education is critical to better delirium care, and wards reported this as the main priority for better care (Fig. 3a), but equally selected lack of time to train staff as one of the most common barriers. Having at least one training session in the preceding year was associated with much higher rates of using a formal assessment tool. It is possible that there are other mediators at play here, such that staff training and using formal tools may both relate to higher prioritisation of delirium at the hospital or ward level, greater staff engagement around delirium, etc. However, the finding does support regular staff training on delirium for all wards as part of the solution to better assessment.

No difference was recorded in non-pharmacological prevention and treatment interventions across the wards, or according to whether there was at least one training session in the preceding year. Mostly, there was a good number of non-pharmacological treatments in use across the wards. This leads us to propose that detecting delirium might be the first and most important care improvement need in Irish hospitals, as personal judgment misses cases [23, 24].

Previous studies presented the primary barriers to delirium care being a lack of time and insufficient knowledge about delirium and its assessment, rather than a lack of awareness of its high prevalence and clinical significance [25–27]. In other words, staff recognized the problem but could not address it within their existing human resources. Mirroring these findings, the most cited barriers to implementation and/or use of evidence-based strategies in our study were lack of time to educate and train staff, shortages of staff, and missing knowledge about delirium, rather than deficient attitudes or unmotivated staff. Moreover, in addition to the presented list, 37.5% of participants who suggested additional barriers cited environmental challenges. This seems to be a considerable concern for several wards and requires further investigation, noting that improving the environment is possible at relatively low cost (better signage, wayfinding, orientation aids, etc.) (INAD-2).

## Strengths and limitations

The current study's strengths include its large sample size, including more than 130 wards from various settings across Ireland. Delirium detection/prevention/management activities and also current barriers to best practice were assessed. Some limitations should be acknowledged. First, participation was on a voluntary basis, with less than half of all acute hospitals participating, risking response bias and a positively skewed dataset [28]. Second, the South-Southwest hospital group was over-represented (many hospitals in this group took part and many wards were included per hospital). This may simply reflect the clinical location of the senior author as a study champion regionally, and this being a relatively large hospital group, but we also note the existence of a specific dementia quality improvement steering committee in this hospital group, the only one of its kind in Ireland at the time of the study, which may have motivated participation. Finally, we did not collect data regarding patients, preventing us from assessing how the patients’ characteristics impact the rate of performance and accuracy of delirium assessments and the overall delirium care plan. Data was reported by ward staff without accuracy checking, which may have led to more favourable results than if an external audit had occurred.

## Conclusion

Our findings suggest that staff training/education in delirium care is a crucial need in Irish hospitals, given its association with higher rates of using a formal tool for delirium assessment, which in turn is associated with fewer missed cases of delirium. Ward staff identified that current barriers to delirium care are not a lack of awareness of the importance of this condition or motivation among staff so much as insufficient staff resources and time to train staff in specific delirium-related skills.

## Appendix

**Table 1:** Full results of the model predicting the use of a formal delirium assessment tool

Predictors	Odds Ratios	CI	p
(Intercept)	0.20	0.06 – 0.65	**0.007**
Receiving at least one educational training about delirium in the last year	8.26	2.09 – 32.54	**0.003**
Flyers for the staff	0.36	0.09 – 1.44	0.149
Delirium being mentioned in handovers	0.31	0.08 – 1.17	0.084
Pocket cards	1.41	0.38 – 5.15	0.606
Informational posters	1.56	0.49 – 4.98	0.451
Knowing that there was a delirium expert on the ward	0.16	0.04 – 0.64	**0.010**
Communication of delirium screening rate	7.05	2.02 – 24.61	**0.002**
Other processes and activities	6.84	1.54 – 30.44	**0.012**
Written protocol for delirium	4.59	1.29 – 16.40	**0.019**
